# Mindfulness-Based Program Plus Amygdala and Insula Retraining (MAIR) for the Treatment of Women with Fibromyalgia: A Pilot Randomized Controlled Trial

**DOI:** 10.3390/jcm9103246

**Published:** 2020-10-11

**Authors:** Juan P. Sanabria-Mazo, Jesus Montero-Marin, Albert Feliu-Soler, Virginia Gasión, Mayte Navarro-Gil, Héctor Morillo-Sarto, Ariadna Colomer-Carbonell, Xavier Borràs, Mattie Tops, Juan V. Luciano, Javier García-Campayo

**Affiliations:** 1 Institut de Recerca Sant Joan de Déu, Esplugues de Llobregat, 08950 Barcelona, Spain; jp.sanabria@pssjd.org (J.P.S.-M.); a.colomer@pssjd.org (A.C.-C.); 2 Teaching, Research & Innovation Unit, Parc Sanitari Sant Joan de Déu, St. Boi de Llobregat, 08830 Barcelona, Spain; 3 Faculty of Psychology, Universitat Autònoma de Barcelona, Bellaterra (Cerdanyola del Vallès), 08193 Barcelona, Spain; xavier.borras@uab.cat; 4 Department of Medicine, International University of Catalonia, C/Josep Trueta s/n, Sant Cugat del Vallès, 08195 Barcelona, Spain; 5 Department of Psychiatry, University of Oxford, Warneford Hospital, Oxford OX3 7JX, UK; jesus.monteromarin@psych.ox.ac.uk; 6 Instituto de Investigación Sanitaria Aragón, Hospital Universitario Miguel Servet, 50009 Zaragoza, Spain; virginia.gasion@gmail.com (V.G.); maytenavarrogil@gmail.com (M.N.-G.); jgarcamp@gmail.com (J.G.-C.); 7 Primary Care Prevention and Health Promotion Research Network, RedIAPP, 28220 Madrid, Spain; 8 Basic Psychology Department, Faculty of Psychology, University of Zaragoza, 44003 Teruel, Spain; hmorillosarto@gmail.com; 9 Developmental and Educational Psychology Unit, Leiden University, 233 AK Leiden, The Netherlands; m.tops@fsw.leidenuniv.nl

**Keywords:** fibromyalgia, mindfulness, amygdala and insula retraining, mind–body techniques, multicomponent intervention, brain-derived neurotrophic factor, immune-inflammatory markers, randomized controlled trial, pilot study

## Abstract

The lack of highly effective treatments for fibromyalgia (FM) represents a great challenge for public health. The objective of this parallel, pilot randomized controlled trial (RCT) was two-fold: (1) to analyze the clinical effects of mindfulness plus amygdala and insula retraining (MAIR) compared to a structurally equivalent active control group of relaxation therapy (RT) in the treatment of FM; and (2) to evaluate its impact on immune-inflammatory markers and brain-derived neurotrophic factor (BDNF) in serum. A total of 41 FM patients were randomized into two study arms: MAIR (intervention group) and RT (active control group), both as add-ons of treatment as usual. MAIR demonstrated significantly greater reductions in functional impairment, anxiety, and depression, as well as higher improvements in mindfulness, and self-compassion at post-treatment and follow-up, with moderate to large effect sizes. Significant decreases in pain catastrophizing and psychological inflexibility and improvements in clinical severity and health-related quality of life were found at follow-up, but not at post-treatment, showing large effect sizes. The number needed to treat was three based on the criteria of ≥50% Fibromyalgia Impact Questionnaire (FIQ) reduction post-treatment. Compared to RT, the MAIR showed significant decreases in BDNF. No effect of MAIR was observed in immune-inflammatory biomarkers (i.e., TNF-α, IL-6, IL-10, and hs-CRP). In conclusion, these results suggest that MAIR, as an adjuvant of treatment-as-usual (TAU), appears to be effective for the management of FM symptoms and for reducing BDNF levels in serum.

## 1. Introduction

Fibromyalgia (FM) is a disabling syndrome of unknown etiology mainly characterized by widespread musculoskeletal pain and symptoms such as fatigue, stiffness, sleep problems, perceived cognitive dysfunction, and distress [1]. FM affects about 2% of the general population worldwide, generating a great economic burden for public health [2]. The lack of curative treatments for FM represents a challenge for clinicians and researchers [3]. The complexity of managing the multiple factors involved in FM and the lack of highly effective treatments have motivated the testing of innovative non-pharmacological therapies in recent years [4]. The scientific evidence compiled to date suggests that multicomponent treatments are the most effective for the management of chronic pain and FM [5,6].

In this scenario, non-pharmacological treatments based on psychoeducation, physical exercise, mindfulness, and cognitive-behavior therapy (CBT) have proven their effectiveness for improving mental health, increasing physical function, decreasing symptoms, and strengthening the acceptance of FM, obtaining small to medium effect sizes [5,6,7,8,9,10,11,12,13,14,15]. Furthermore, multicomponent treatments integrating some of these practices have been considered the gold-standard for managing FM [6,12]. Meta-analyses have provided evidence that mindfulness-based interventions (MBIs) are especially effective for improving quality of life and pain compared to usual care and even some active control treatments [11,12]. In addition, MBIs have been shown to be effective in modifying FM-related immune-inflammatory markers [13]. Specifically, amygdala and insula retraining (AIR), a mind–body approach, has preliminarily demonstrated improvements in physical health, energy, pain, distress, and fatigue in patients with FM and chronic fatigue syndrome (CFS) [16]. AIR was originally designed for patients with CFS [17] as a method of hypothetically reducing chronic over-sensitization and heightened fear response of the amygdala which may underlie some of the symptoms related to both CFS and FM [17,18]. These specific techniques aim to retrain conditioned somatic signaling in the brain which may keep the nervous system and the immune system in a state of heightened arousal. This is achieved through specific and specialized interventions that seek to strengthen neurological inhibitory mechanisms in areas of the prefrontal and orbital cortices, the insula, and the anterior and posterior cingulate [16,17,18]. Retraining techniques involve repeatedly interrupting signals from the amygdala and insula using a variety of generative practices tailored to the individual patient [19]. The patient is taught to become aware of the internal signals of the symptoms, and then to act on these signals in a specific way that drastically interrupts the signaling. At the same time, mindfulness training has also been shown to reduce amygdala reactivity, and to increase grey matter volume in the prefrontal cortex and the insula [20], thus its potential combination with AIR to further increase its effects is appealing.

Pain processing is facilitated by complex neural networks (i.e., pain matrix) involving perception, cognition, and emotion [21] with all these processes orchestrated by a group of brain structures jointly activated by painful stimuli. The pain matrix includes amygdala which has a nuclear role in conditioning the learning processes that occur during aversive and traumatic experiences. Additionally, insular cortex (along with other cortical areas such as the anterior cingulate and medial prefrontal cortex) also plays a relevant role in the high-order control of amygdala-mediated fear-conditioning [17]. Interestingly, both structures seem to present morphological [22] and functional [23,24] abnormalities in patients with FM correlating with pain intensity and other FM symptoms [25]. At the same time, there is mounting evidence that immune-inflammatory abnormalities in patients with FM (e.g., increased levels of pro-inflammatory cytokine IL-6, decreased levels of anti-inflammatory IL-10) may also contribute to the abnormal processing of pain signals (both the CNS and peripheral nervous system [PNS]), promoting and maintaining FM symptomatology [13]. Besides, increased levels of brain-derived neurotrophic factor (BDNF) have also been found in FM, with some authors suggesting its potential implication in pain chronification [26] as it plays a key role in a variety of neuroplasticity processes, including pain modulation, pain transduction, nociception, and hyperalgesia [27]. Anti-inflammatory effects (e.g., reductions in C-reactive protein) of mind–body therapies have been reported previously [13,28,29] and decreases in BDNF levels after another mind–body therapy (i.e., attachment-based compassion therapy) followed by improvements in functional impairment in patients with FM have also been previously observed [29]. Altogether, this suggests a potential biological path driving part of the benefits of mind–body interventions in FM.

Taking this state of the question as its foundation, the objective of this 8 week parallel, pilot randomized controlled trial (RCT) was two-fold: (1) to analyze the clinical effects of mindfulness plus amygdala and insula retraining (MAIR) compared to a structurally equivalent active control group of relaxation therapy (RT; [19]) in the treatment of FM; and (2) to evaluate its impact on immune-inflammatory markers and BDNF in serum. Our general exploratory hypotheses where that: (a) MAIR would result in significantly superior improvements in primary (i.e., FM functional impact) and secondary clinical variables compared with RT; and (b) MAIR would be related with decreases in pro-inflammatory markers and BDNF levels as well as significantly greater increases in the anti-inflammatory markers in comparison to RT.

## 2. Experimental Section

### 2.1. Research Design

An 8 weeks parallel pilot RCT with two arms (MAIR vs. RT) and assessment periods at baseline, post-intervention, and a 3 month follow-up. This work reports secondary data embedded in a larger RCT (ClinicalTrials.gov Registration: NCT02454244) [30,31]. The RCT was conducted following the “Consolidated Standards of Reporting Trials” (CONSORT) guidelines [21] and the “Initiative on Methods, Measurement and Pain Assessment in Clinical Trials” (IMMPACT) recommendations [32].

### 2.2. Study Sample

Potential participants were recruited from eight primary healthcare centers in Zaragoza (Spain). The inclusion criteria were: (1) age between 18 and 65 years; (2) having an FM diagnostic in accordance with the American College of Rheumatology (ACR) 1990 criteria and provided by a rheumatologist working for the Spanish National Health Service; and (3) being able to read and understand Spanish. The exclusion criteria were: (1) the presence of severe Axis I psychiatric/somatic disorder, autoimmune disease, or the use of corticosteroid medication; and (2) participation in a concurrent RCT. As long as the patients agreed not to change their medication dosage during the RCT period, they were not asked to discontinue their regular pattern of medication which was considered part of their usual care. Medication was maintained stable 3 months prior the study and throughout the study.

The sample size was estimated by considering a clinically relevant expected difference of ≥20% in the FM functional status (i.e., score in Fibromyalgia Impact Questionnaire (FIQ)). A previous study with a similar sample reported a mean FIQ score of 70.8 (SD = 15.2) [33]. Therefore, a between-groups difference of 14.2 points was established as a target for the present study (equivalent to 0.95 SDs). To detect this difference with an overall α level at 5% and a statistical power set at 80% in two-tailed tests, we needed 18 patients per group. Since we expected a dropout rate of around 15% [30], we increased the sample size to reach the number of 41 participants at baseline.

### 2.3. Procedure

**Figure 1:**
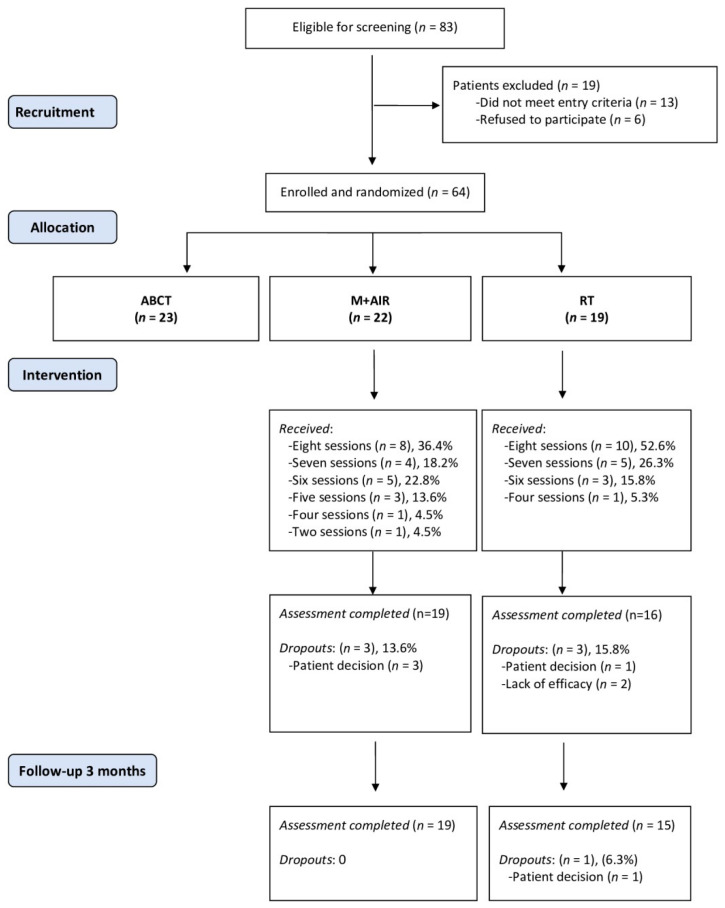
Flowchart of participants in the pilot Randomized Controlled Trial (RCT). Note: The results of the ABCT arm were detailed in Montero-Marín et al. (2018) [30]. ABCT: attachment-based compassion therapy; MAIR: mindfulness plus amygdala and insula retraining; RT: Relaxation Therapy. More details regarding the ABCT arm can be found in Montero-Marin et al. [29].

General practitioners (GPs) identified potential patients during routine consultations who were then re-evaluated in a face-to-face interview with an external researcher until achieving the required sample size. Patients who met all inclusion/exclusion criteria and provided their written informed consent were then randomized. An independent researcher generated a random group allocation sequence by using a computer software. The group assignment was informed via telephone after the completion of baseline assessments, and the allocation details were concealed from the rest of researchers of the study until all patients had been assigned. Participants were not informed about which treatment was the target of the RCT in order to reduce a potential expectancy effect. The outcome assessor remained blind to patient allocation (Figure 1). Interventions were held in Health Public Center Arrabal, Zaragoza. MAIR groups were conducted by a health psychologist with more than 5 years of experience in AIR and mindfulness training. RT groups were also conducted by a health psychologist with comparable experience in relaxation training.

Patients were scheduled for blood extraction before starting the intervention and within 5 days following its completion. To minimize circadian variability in immunological markers, all blood samples were collected between 8:00 and 8:30 a.m., after night fasting. Upon the completion of the extraction, blood was centrifuged and serum was obtained and then frozen to −80 °C until the biochemical analyses at LABCO laboratories (Barcelona, Spain). The parallel design of the study ensured that the groups would display equivalent seasonal variability. A battery of measures was administered to the patients in both study arms at pre-treatment, post-treatment, and the 3 months follow-up. Biomarkers were measured in both groups at pre-treatment and post-treatment.

The study protocol (PI15/0049; 01/04/2015) was approved by the ethical review board of the regional health authority of Aragon (CEICA), Spain. A full explanation of the procedure can be found elsewhere [29].

### 2.4. Treatments

#### 2.4.1. Relaxation Therapy (RT)

The active control group completed a relaxation program consisting of eight weekly 2 h sessions followed by three monthly sessions. RT was based on the four techniques described by Montero-Marín et al. [29]: (1) *visualizations*, (2) *autogenic relaxation*, (3) *progressive relaxation*, and (4) *breathing* (Table 1). The program encourages personal practices by providing daily homework assignments of around 15–20 min. The therapist was a clinical psychologist with accredited expertise in relaxation techniques. This treatment was added to TAU. MAIR and RT were structurally equivalent, which aimed to control for non-specific factors.

#### 2.4.2. Mindfulness + Amygdala and Insula Retraining (MAIR)

Patients completed some practices included in the mindfulness-based stress reduction (MBSR) program that were added to the amygdala and insula retraining techniques (AIR) [17]. This psychotherapeutic approach focused on improving skills and strategies for coping with stressful situations by hypothetically interrupting the conditioned responses of anxiety or fear from the amygdala. It is composed of psychological techniques such as breathing, meditation, and neurolinguistic programming (Table 1). MAIR consisted of eight weekly 2 h sessions followed by three monthly sessions. Similar to the RT program, MAIR also includes daily homework assignments that take approximately 15 to 20 min to complete. The therapist was a psychologist with accredited training in MBSR and AIR. This treatment was added to the TAU.

### 2.5. Measures

#### 2.5.1. Socio-Demographic Characteristics

Patients completed a socio-demographic survey at baseline including age, gender, marital status, dwelling, place of residence, education, and employment status.

#### 2.5.2. Primary Outcome

The Fibromyalgia Impact Questionnaire (FIQ) is the gold-standard self-reporting measure for assessing the functional impact of FM. It is based on 20 items capturing a broad spectrum of symptoms and difficulties related to FM. Scores range from 0 to 100, with greater scores indicating a higher functional impact of FM. The Spanish version of the FIQ has shown good psychometric properties [34].

#### 2.5.3. Secondary Outcomes

The Clinical Global Impression-Severity Scale (CGI-S) is a 7-point tool asking for a clinician’s perception of severity regarding a patient’s specific disease. Scores range from 1 to 7, with greater scores indicating higher clinical severity. CGI-S is one of the most used brief assessment instruments, and it has been administered to FM patients in previous studies (e.g., [35]).

The Pain Catastrophizing Scale (PCS) is a 13-item self-reporting measure that measures pain catastrophizing, and comprises three dimensions: rumination, magnification, and helplessness. Scores range from 0 to 52, with greater scores indicating higher pain catastrophizing. The Spanish adaptation of the PCS presented adequate psychometric properties in patients with FM [36].

The Hospital Anxiety and Depression Scale (HADS) is a self-reporting questionnaire that assesses the severity of anxiety and depressive symptoms by using 14 items on a four-point Likert-type scale. The HADS comprises two subscales: anxiety (HADS-A) and depression (HADS-D), each score ranging from 0 to 21, with greater scores indicating a higher severity of symptoms (anxiety and depression). The Spanish version of the HADS demonstrates adequate psychometric properties in patients with FM [37].

The Visual Analogue Scale (VAS) from the EuroQol instrument (EQ-VAS) asks the patient to rate their perceived current health status in a line from the best to worst possible health states, from 100 or 0 points, respectively. The Spanish version of the EQ-5D is a reliable and valid outcome measure in clinical trials [38].

The Acceptance and Action Questionnaire (AAQ-II) is a seven-item instrument assessing psychological inflexibility. Scores range from 7 to 49, with greater scores indicating higher psychological inflexibility. The Spanish version of the AAQ-II has good psychometric properties [39].

The Five Facets of Mindfulness Questionnaire (FFMQ) is a 39-item self-reporting measure that evaluates mindfulness. The mindfulness facets assessed are: (1) observing, (2) describing, (3) acting with awareness, (4) non-judging of inner experiences, and (5) non-reacting. A total score can be calculated by summing item scores. The Spanish version of the FFMQ shows good psychometric properties [40].

The Self-Compassion Scale (SCS) is a 26-item measure designed to evaluate overall self-compassion considering the facets of common humanity, mindfulness, and self-kindness assessed in six subscales. Scores range from 6 to 30, with greater scores indicating higher self-compassion. The Spanish version of the SCS showed adequate psychometric properties [41].

Treatment credibility (on a scale from 0 to 10) and the patient’s preferred choice of intervention (i.e., MAIR, RT, other, indifferent) were assessed before group assignment. All sessions were audio recorded, and two of the researchers (J.G.-C. and H.M.-S.), randomly assessed two sessions of each treatment condition to confirm that the psychological interventions faithfully followed the corresponding protocol.

#### 2.5.4. Biomarkers Outcomes

BDNF was analyzed with ELISA (R&D systems©), Interleukin (IL)-6, TNF-α, and IL-10 with Immulite© 1000 (Siemens), and high sensitivity C-Reactive Protein (hs-CRP) with immunoturbidimetry (CRP Beckman Coulter©). All analyses were conducted in accordance with the manufacturer instructions. All biomarkers were obtained from serum.

### 2.6. Data Analyses

All statistical analyses were computed using SPSS v.25. Baseline between-group differences in sociodemographic, clinical variables, and biomarkers were evaluated, applying the Student *t*-test, for continuous variables, and the χ^2^-test, for categorical data (Fisher’s test was used when adequate). All outcome measures were evaluated for normality with the Kolmogorov–Smirnov test. Levels of inflammatory biomarkers were subjected to a natural logarithmic transformation to normalize the significantly skewed data distributions (clinical variables scores and BDNF concentrations were distributed normally, so they were analyzed without any transformation).

Analyses were performed with hierarchical linear mixed-effects models, nesting participants at level 1 as random effects to control for the correlation between repeated measurements, and considering each outcome variable, on an intention-to-treat (ITT) basis. Statistical analyses for clinical outcomes included pre, post and 3 month follow-up data; data on biomarkers were only available for pre- and post-intervention assessments. Restricted maximum likelihood regression (REML) was used to account for the correlation between the repeated measures for each individual; this approach produced less biased estimates of variance parameters when using small sample sizes or unbalanced data [42]. Regression coefficients (B) and 95% confidence intervals (95% CI) were calculated for the Group × Time interaction between the groups at post-intervention and follow-up assessments. For each pairwise comparison, Cohen’s d (0.20 = small, 0.50 = medium, and 0.80 = large effect size) was calculated by using the pooled SD at baseline to weigh the differences in the pre–post means, and to correct for the population estimate [43]. The total number of medications was included as a covariate in all analyses for testing a potential effect on the results. We also applied Benjamini–Hochberg correction for multiple comparisons, a procedure to detect false discovery designed to overcome the limitations that other commonly used tests have shown [44].

We classified patients into responders/non-responders by using two different cut-off criteria: (a) ≥20% reduction in the pre–post FIQ total score [45]; and (b) ≥50% reduction in the pre–post FIQ total score [32]. These two classifications were used to compute the number needed to treat (NNT) in MAIR compared with the RT control group. NNT is an index which allows findings from RCTs to be more meaningful to clinicians and refers to the estimated number of patients who need to be treated with the new proposed treatment (i.e., rather than the comparison intervention) for one additional patient to benefit (to be a “responder”, in this case). A 95% confidence interval (CI) for each NNT was calculated.

## 3. Results

### 3.1. Patients Flow and Compliance

Of the 83 potential patients who were eligible, 13 were excluded for not meeting the screening criteria and six refused to participate. The 64 patients enrolled were randomized into three study arms, with 23 patients for ABCT, 22 for MAIR, and 19 for RT (Figure 1). As indicated above, in this study we specifically explored the results of the 41 patients in the MAIR and RT groups. The majority of participants completed the post-intervention clinical assessment (35 of 41, 85.37%); blood extractions at baseline and at post-intervention were obtained from all participants for the biomarkers evaluation. The median number of sessions attended in the MAIR was 7 (Q1 = 6, Q3 = 8), while in the RT it was 8 (Q1 = 7, Q3 = 8), which was not a statistically significant difference (*p* > 0.05). The retention rate for the MAIR was 86.36% and 86.36% at post-treatment and follow-up, respectively; and for RT this was 84.21% and 78.94%, respectively. The ratio of dropouts was very similar in the two arms, both at post-treatment (*p* > 0.05) and at follow-up (*p* > 0.05).

### 3.2. Baseline Socio-Demographic and Clinical Characteristics of Patients

The socio-demographic and baseline clinical features of the patients allocated in each treatment condition are shown in Table 2. The patients were women in their early fifties, mostly with a stable partner, and dwelling in their own home in an urban residence. In clinical terms, the patients presented moderate severity [46] regarding their functional status (FIQ:M = 67.00 (SD = 17.98)). Part of the patients were under medication (see Table 2 for more details). There were no significant differences between the groups for any of the referenced variables.

### 3.3. Effects on Primary and Secondary Outcomes

Table 3 shows the descriptive statistics and between-group analyses for the primary and secondary clinical outcomes after controlling for the total number of medications. For the primary outcome, MAIR was significantly superior to RT for reductions in the functional impact (FIQ) at post-treatment and follow-up, both with large effect sizes.

Regarding the secondary outcomes, the significantly moderate effect sizes of MAIR in comparison to RT were observed. We found significant decreases in the moderate effect size in clinical severity (CGI-S) as well as large effects in pain catastrophizing (PCS), both at follow-up, but not at post-treatment. Compared to RT, MAIR showed a significant improvement in anxiety (HADS-A) and depression (HADS-D) at post-treatment and follow-up, all with moderate effect sizes. Regarding the effects of MAIR on perceived health (EQ-VAS), a significant effect was found only at follow-up. Significant and large effect sizes of MAIR were obtained for a reduction in psychological inflexibility (AAQ-II) at follow-up, but again no difference was found at post-treatment. Moderate-to-large effect sizes in self-compassion (SCS) and mindfulness scores (FFMQ) were observed at post-treatment and at follow-up.

### 3.4. Effects on Biomarkers

As seen in Table 4, a significant moderate effect size of MAIR in comparison to RT was observed in the BDNF levels at post-treatment after controlling for the total number of medications. There were no significant differences between the groups in hs-CRP, TNF-α, IL-6, and IL-10 at post-treatment.

### 3.5. Absolute Risk Reduction and Number Needed to Treat (NNT)

First, 84.2% and 18.8% of the patients in the MAIR and RT (16 of 19, and 3 of 16, who completed pre- and post-treatment), respectively, reached the criterion of ≥20% FIQ reduction after treatment. Therefore, the probability of success in MAIR compared to RT increased by 65.5% (95% CI = 40.3% to 90.7%), with an NNT = 2 (95% CI = 1.1% to 2.5%). Second, 36.8% and 0% of the patients in the MAIR and RT (7 of 19, and 0 of 16, who completed the pre- and post-treatment), respectively, reached the criterion of ≥50% FIQ reduction after treatment. Thus, the absolute risk reduction in MAIR compared to RT increased by 36.8% (95% CI = 15.2% to 58.5%), with an NNT = 3 (95% CI = 1.7% to 6.6%).

### 3.6. Patient Preferences and Credibility of Therapies

The preferred intervention of each patient was evaluated before randomly allocating to interventions, and a similar distribution was observed between the arms. Specifically, eight (42.1%) patients in the RT and 10 (45.5%) in the MAIR had no specific preference. Each patient rated the credibility of their assigned intervention after receiving it (scores ranging from 0—minimum credibility, to 10—maximum credibility), and similar values were found between the groups in this regard (MAIR, *median* = 8, (*Q*_1_ = 8, *Q*_3_ = 9); RT, *median* = 8, (*Q*_1_ = 7, *Q*_3_ = 9)).

## 4. Discussion

In line with previous literature testing MBIs for FM (e.g., [14]) and AIR [16,17], this pilot study showed that MAIR, as an add-on to TAU, is an efficacious intervention—with moderate-to-large effect sizes—for improving a wide range of outcomes: functional impairment, clinical severity, and quality of life along with the cognitive processes associated to psychopathology, such as mindfulness, and self-compassion. The beneficial effects of MAIR remained significant in the 3 month follow-up assessment and even improved in terms of clinical severity, perceived health, pain catastrophizing and psychological flexibility. Additionally, a significant reduction in BDNF levels was observed in the MAIR group at the post-intervention evaluation. However, no significant effect of MAIR on cytokine and hs-CRP levels was detected. Furthermore, both MAIR and RT groups showed high credibility (8 out of 10 points) as stated by the patients allocated to each treatment arm. Our findings provided partial support to our hypotheses since, although superior clinical improvements and greater BDNF reductions were found in MAIR compared to RT, no significant changes were found regarding immune-inflammatory variables.

We observed an overall significant pre–post decrease in FIQ scores (i.e., 37% of reduction) in the MAIR group, with a large effect size. Compared to the results obtained by Toussaint et al. [16] in patients with FM allocated to a “pure” amygdala retraining group, where a more modest overall improvement (24% reduction on FIQ scores) was observed, our results suggest a potential superior effect of the combination of mindfulness and AIR. However, a comparison between both studies is only tentative since MAIR was a 2 month on-site therapy while amygdala retraining in Toussaint et al.’s study [16] had only 2.5 h of on-site teaching plus one month of a video-based home course and a very small sample (*n* = 7). Interestingly, the effect sizes of the pre–post changes in the MAIR group were even greater than those observed after the MBIs of equivalent duration (e.g., [14]). The positive effects of MAIR were also observed in a wide range of clinical measures and salutary cognitive variables such as mindfulness, psychological flexibility and self-compassion. Remarkably, these benefits were maintained or even improved at the 3 month follow-up, especially in the case of pain catastrophizing and psychological inflexibility that are known to be crucial cognitive elements in explaining the impact of the syndrome [47]. Further dismantling studies including MAIR and structurally equivalent interventions of “pure” AIR and mindfulness training may allow to evaluate if a potential synergic effect exists between both interventions.

Although some studies have reported significant changes in immune-inflammatory biomarkers after non-pharmacological interventions in FM [48], MAIR did not impact on pro- or anti-inflammatory cytokines or hs-CRP levels (although a downward trend for hs-CRP and IL-10 was observed). In this regard, our results resemble those in which pro-inflammatory markers (i.e., IL-6, TNF-α) remained stable in the mindfulness group in comparison with the control group [13]. However, in the study of Andrés-Rodríguez et al. [13] a positive regulatory effect of the mindfulness intervention on the levels of the anti-inflammatory cytokine IL-10 was observed which we did not. A recent systematic review and meta-analysis [49] has brought into question the hypothesis of a disbalance between pro- and anti-inflammatory cytokines in FM [50] so a lack of effects of MAIR on inflammatory biomarkers may simply rely on a bottom/ceiling-effect.

Nevertheless, decreases in BDNF levels (moderate effect size) were found in the MAIR group. BDNF is known to play a crucial role in a variety of neuroplasticity processes, including pain modulation, pain transduction, nociception, and hyperalgesia [27], all of which were altered in FM. Moreover, some studies have also suggested that FM and other central sensitivity syndromes may particularly present abnormalities in biomarkers related to neuronal plasticity, such as BDNF [51]. In this regard, increased plasma levels of BDNF have been reported in patients with FM [52]. However, divergent results have been obtained regarding the role of BDNF in FM, with studies finding a lack of association between BDNF and patients’ clinical complaints [53,54] or finding comparable levels between FM and healthy subjects [54]. Significant pre–post decreases in BDNF observed in the MAIR group are in agreement with those observed in other effective cognitive–behavioral third-wave interventions such as attachment-based compassion therapy [29] and after a 2-week thermal therapy program [55]. It is also worth mentioning that low serum levels of BDNF have been found to be a characteristic of depression [56]; in this regard, one could expect that after an intervention with a positive effect on depressive symptoms (as it was the case of MAIR), increases in BDNF levels should be observed. However, we did not find such increases as a significant decrease in the levels of this biomarker was found after MAIR. Our findings may be partially explained by the fact that the study sample showed mild depressive symptoms at baseline (with mean depression scores based on HADS around the minimum cut-off point for caseness of 8 points [57]), thus patients having a major depressive disorder should be a minority in our study. Furthermore, research showed that changes in the serum levels of BDNF during antidepressant treatments are not always in parallel to clinical improvements (i.e., severity of depressive symptoms) [56]. Finally, the BDNF levels of the MAIR group at post-test approached those of pain-free controls in the referenced study [51], altogether pointing towards a normalizing effect of MAIR in the BDNF levels. In summary, this present study provides additional support to those suggesting that BDNF is involved in the pathophysiology of some abnormal pain syndromes such as FM [52], and informs of a new potential non-pharmacological treatment (i.e., MAIR) with a normalizing effect on BDNF levels, potentially reducing the dysfunctional neuroplastic processes behind FM.

There are some potential limitations in this pilot study that should be acknowledged when interpreting the effects of treatment. First, while the final sample size used was within the limits of the power calculation, it was small to generate strong conclusions. Second, the possible influences of therapist variables were not controlled, making it impossible to recognize their effect on treatment. In this regard, further studies should also consider randomizing therapists between intervention groups. Third, the intervention was not compared with other psychological treatments that have demonstrated effectiveness for FM (e.g., CBT or acceptance and commitment therapy (ACT)) or with an inactive control group alone. Fourth, the two treatment components (mindfulness and amygdala and insula retraining) were not evaluated independently, and therefore their specific effects with respect to the combination could not be compared. Furthermore, it was not possible to follow-up the levels of the biomarkers. Finally, future studies should also evaluate the effects of MAIR in patients with FM diagnosed according to updated ACR criteria.

To sum up, the lack of curative treatments for FM represents a great challenge for public health. MAIR (compared to an active control group) demonstrated to be an innovative and effective treatment for improving several outcomes in patients with FM, as well as for increasing mindfulness and self-compassion. Furthermore, improvements in all evaluated variables were also observed at 3 month follow-up, with even larger effect sizes compared to post-intervention assessment. Although no effect of MAIR was observed for immune-inflammatory biomarkers (i.e., TNF-α, IL-6, IL-10, and hs-CRP), reductions in serum BDNF levels were observed as being suggestive of a normalizing effect of the intervention on the levels of this neuroplastic agent. The short- and mid-term positive results of MAIR reported in the present study—although exploratory and preliminary in nature—may lead to methodologically sounder RCTs on the effects of MAIR in patients with FM. Furthermore, our results strengthen a line of research on the effects of non-pharmacological therapies on biological variables potentially underpinning core clinical aspects in FM.

## Figures and Tables

**Table 1:** Session outlines of the RT and MAIR.

Session	RT	MAIR
1	*Visualizations I.* Presentation of the different relaxation techniques and their usefulness.	*General overview.* Theoretical aspects of the brain, the limbic system, fear, conditioning and reconditioning. Visualization of 100% recovery.
2	*Visualizations II.* Deepening in guided relaxation through imagination training. Noticing the effects of relaxation in the body and mind and learning when to use it.	*Developing FM.* How stress triggers the nervous system. Mindfulness and self-awareness. Anchoring presence in the body and breathing.
3	*Visualizations III*. Working with emotions through imagination. Emotional burdens are symbolically released, reducing emotional discomfort.	*Amygdala technique.* Reconditioning. Breaking negative thoughts, meanings, and emotions, and somatic answers in the body. Breathing and meditation.
4	*Autogenic relaxation I.* Autogenic relaxation initiation. Fostering sensations of relaxation through imagining a ball of light and heat.	*Regulation of negative emotions and symptoms.* The “soften and flow” self-regulation through mindfulness practice. Body scan meditation.
5	*Autogenic relaxation II.* Deepening in autogenic relaxation by learning when and how to use it. Working on heaviness sensations.	*The accelerator of MAIR*. Internal dialogue, patterns and behaviors related to illness in FM. Importance of mindfulness as a daily practice. Walking meditation.
6	*Progressive relaxation.* Initiation to the progressive relaxation. Tensing and relaxing the muscles to become aware of the change in body sensations.	*Dealing with stress.* Awareness of negative thoughts related to external stimuli. Over-reactions of the nervous system. Mindfulness in daily activities.
7	*Breathing I.* Learning to use breathing exercises. Knowing its benefits. Deep inspiration and exhalation. Using breathing to calm anxiety.	*Awareness of limiting beliefs*. How to identify and change them through reconditioning. Motivation and sense of life. Meditation on values.
8	*Breathing II.* Deepening in breathing exercises by learning different deep-breathing exercises.	*Recovery, cycles, and stages and returning to regular life*. Fear of failure in terms of recovery. Positive visualizations of the future. Review and summary of the protocol.

RT: relaxation therapy; MAIR: mindfulness plus amygdala and insula retraining.

**Table 2:** Baseline socio-demographic and clinical characteristics of study participants.

Characteristics at Baseline	RT (*n* = 19)	MAIR (*n* = 22)	(*p*)
Socio-demographics			
*Sex, female*	19 (100)	22 (100)	−
*Age*	52.21 (5.95)	52.77 (13.45)	0.86
*Marital status, stable relationship*	13 (68.4)	12 (54.5)	0.39
*Residence, urban*	19 (100)	22 (100)	0.27
*Dwelling, own home*	17 (89.5)	18 (81.8)	0.76
Education			0.66
*Primary*	4 (21.1)	7 (31.8)	
*Secondary*	8 (42.1)	8 (36.4)	
*University*	7 (36.8)	7 (31.8)	
Employment			0.43
*Employed*	5 (26.2)	3 (13.6)	
*Sick leave/inability*	8 (42.2)	8 (36.4)	
*Unemployed*	6 (31.6)	11 (50.0)	
Clinical measures			
*Fibromyalgia impact*			
FIQ (0–100)	62.83 (18.41)	70.61 (17.21)	0.17
*Clinical severity*			
CGI-S (1–7)	4.32 (1.16)	4.59 (1.14)	0.45
*Pain catastrophizing*			
PCS (0–52)	25.00 (10.92)	29.50 (9.73)	0.17
*Anxiety and depression*			
HADS-A (0–21)	11.37 (5.40)	12.32 (3.48)	0.52
HADS-D (0–21)	8.05 (6.03)	9.73 (5.19)	0.35
*Perceived health*			
EQ-VAS (0–100)	54.00 (20.19)	48.18 (17.01)	0.32
*Psychological inflexibility*			
AAQ-II (10–70)	37.32 (13.34)	41.00 (10.45)	0.33
*Mindfulness facets*			
FFMQ (39–195)	118.74 (14.96)	116.32 (18.48)	0.65
*Self-compassion*			
SCS (6–30)	16.81 (4.13)	16.63 (3.81)	0.89
Taking pharmacological treatment			
*Non-opioid analgesics*	2 (10.5)	3 (13.6))	0.76
*Opioids*	2 (10.5)	4 (18.2)	0.49
*NSAIDs*	2 (10.5)	2 (9.1)	0.88
*Antidepressants*	2 (10.5)	8 (36.4)	0.06
*Anticonvulsants*	2 (10.5)	4 (18.2)	0.49
*Benzodiazepines*	6 (31.6)	5 (22.7)	0.52
*Antipsychotics*	0 (0.0)	1 (4.5)	0.35
Total number of medications	0.84 (0.96)	1.23 (0.97)	0.21

FIQ: Fibromyalgia Impact Questionnaire; CGI-S: Clinical Global Impression Severity Scale; PCS: Pain Catastrophizing Scale; HADS-A: Hospital Anxiety and Depression Scale-Anxiety; HADS-D: Hospital Anxiety and Depression Scale-Depression; EQ-5D-VAS: Visual Analogue Scale from EuroQol; AAQ-II: Acceptance and Action Questionnaire. FFMQ: Five Facets of Mindfulness Questionnaire; SCS: Self-Compassion Scale; NSAIDs: non-steroidal anti-inflammatory drugs.

**Table 3:** Descriptives and analysis of the primary and secondary clinical variables.

	RT (*n* = 15) M (SD)	MAIR (*n* = 19) M (SD)	*d*	B (95% CI)	*z* (*p*)
*FIQ* (0–100)					
Baseline	61.12 (20.21)	68.03 (17.02)			
Post-treatment	61.22 (25.90)	42.84 (20.57)	−1.34	−26.38 (−40.87–−11.89)	**−3.57 (<0.001)**
Follow-up	67.82 (17.77)	51.05 (16.30)	−1.25	−23.99 (−38.64–−9.33)	**−3.21 (0.001)**
*CGI-S* (1–7)					
Baseline	4.27 (1.28)	4.47 (1.12)			
Post-treatment	4.33 (0.82)	3.79 (0.86)	−0.62	−0.72 (−1.52–0.08)	−1.76 (0.078)
Follow-up	4.07 (0.80)	3.32 (1.00)	−0.79	−0.98 (−1.79–−0.18)	**−2.39 (0.017)**
*PCS* (0–52)					
Baseline	25.93 (10.14)	30.13 (8.40)			
Post-treatment	23.47 (14.49)	22.67 (13.14)	−0.52	−3.48 (−10.07–3.12)	−1.03 (0.302)
Follow-up	23.53 (13.58)	16.20 (9.83)	−1.20	−10.00 (−16.41–−3.59)	**−3.06 (0.002)**
*HADS-A* (0–21)					
Baseline	11.53 (6.06)	12.42 (3.73)			
Post-treatment	10.53 (5.24)	8.05 (3.60)	−0.68	−3.15 (−5.37–−0.93)	**−2.78 (0.005)**
Follow-up	9.80 (4.84)	6.84 (1.54)	−0.78	−3.75 (−6.00–−1.50)	**−3.27 (0.001)**
*HADS-D* (0–21)					
Baseline	8.33 (6.67)	9.32 (5.11)			
Post-treatment	7.53 (4.81)	5.05 (3.70)	−0.59	−4.07 (−6.54–−1.60)	**−3.23 (0.001)**
Follow-up	7.80 (5.99)	5.47 (3.57)	−0.56	−3.66 (−6.16–−1.15)	**−2.86 (0.004)**
*EQ-VAS* (0–100)					
Baseline	53.07 (21.71)	47.89 (16.10)			
Post-treatment	56.87 (18.95)	64.74 (16.87)	0.69	12.26 (−0.55–25.08)	1.88 (0.061)
Follow-up	61.67 (15.66)	70.63 (14.29)	0.75	14.08 (1.11–27.04)	**2.13 (0.033)**
*AAQ-II* (10–70)					
Baseline	38.00 (14.23)	40.95 (11.20)			
Post-treatment	39.07 (13.90)	34.79 (11.65)	−0.57	−7.64 (−15.43–0.15)	−1.92 (0.055)
Follow-up	37.00 (12.78)	26.21 (4.74)	−1.08	−14.06 (−21.95–−6.18)	**−3.49 (<0.001)**
*FFMQ* (39–195)					
Baseline	120.07 (16.36)	117.16 (18.41)			
Post-treatment	121.87 (23.71)	131.79 (17.95)	0.71	13.44 (2.87–24.01)	**2.49 (0.013)**
Follow-up	122.67 (19.88)	132.32 (13.06)	0.70	12.46 (1.76–23.16)	**2.28 (0.022)**
*SCS* (6–30)					
Baseline	17.23 (4.40)	16.31 (3.64)			
Post-treatment	17.19 (4.57)	20.31 (4.25)	0.99	3.80 (1.45–6.15)	**3.17 (0.002)**
Follow-up	17.14 (4.53)	23.28 (3.35)	1.73	6.78 (4.40–9.16)	**5.58 (<0.001)**

Note: RT: relaxation therapy; MAIR: mindfulness plus amygdala and insula retraining; M: mean; SD: standard deviation; *d*: Cohen’s *d* effect size corrected for repeated measures; B: unstandardized regression coefficient; 95% CI: 95% confidence interval; FIQ: Fibromyalgia Impact Questionnaire; CGI-S: Clinical Global Impression Severity Scale; PCS: Pain Catastrophizing Scale; HADS-A: Hospital Anxiety and Depression Scale-Anxiety; HADS-D: Hospital Anxiety and Depression Scale-Depression; EQ-5D-VAS: Visual Analogue Scale from EuroQol; AAQ-II: Acceptance and Action Questionnaire. FFMQ: Five Facets of Mindfulness Questionnaire; SCS: Self-Compassion Scale. When the Benjamini–Hochberg correction was applied to correct for multiple comparisons, all significant effects were maintained. Results show models adjusting for the “total number of medications” as a covariate. Total number of medications was a significant covariate (*p* < 0.05) in all analyses with the exception of EQ-VAS (*p* = 0.052), AAQ-II (*p* = 0.073) and SCS (*p* = 0.244). Bold: significant values.

**Table 4:** Analyses of serum biomarkers.

	RT (*n* = 16) M (SD)	MAIR (*n* = 19) M (SD)	*d*	B (95% CI)	*z* (*p*)
BDNF					
Pre-	19.34 (6.62)	22.72 (8.24)			
Post-	21.54 (7.08)	20.47 (6.13)	−0.58	−5.94 (−9.65–−2.23)	**−3.13 (0.002)**
hs-CRP					
Pre-	3.54 (4.36)	4.68 (6.42)			
Post-	4.00 (4.12)	3.85 (5.50)	−0.23	−0.54 (−1.19–0.11)	−1.64 (0.101)
TNF-α					
Pre-	5.99 (2.74)	5.92 (1.65)			
Post-	5.93 (4.13)	5.60 (2.30)	−0.12	−0.06 (−0.25–0.12)	−0.68 (0.499)
IL-6					
Pre-	3.04 (1.18)	3.35 (2.63)			
Post-	3.14 (1.87)	3.44 (1.12)	−0.01	0.12 (−0.24–0.49)	0.65 (0.514)
IL-10					
Pre-	5.13 (0.43)	5.59 (1.44)			
Post-	5.02 (1.41)	5.31 (0.53)	−0.15	−0.07 (−0.16–0.02)	−1.51 (0.132)

Note: M: mean. SD: standard deviation. *d*: Cohen’s *d*. B: regression coefficient. 95% CI: 95% confidence interval. When the Benjamini–Hochberg correction was applied to correct for multiple comparisons, all significant effects were maintained. Results show models adjusting for the “total number of medications” as a covariate. The total number of medications was not a significant covariate (*p* > 0.05) in any model. Bold: significant values.
